# A Pragmatic Low-Cost Digital Support Pathway for GDMT Optimization in Ambulatory HFrEF: An Exploratory 6-Month Matched Cohort Study

**DOI:** 10.3390/healthcare14121675

**Published:** 2026-06-12

**Authors:** Miruna Popovici, Nilima Rajpal Kundnani, Marius Papurica, Anca-Raluca Dinu, Victor Buciu, Ovidiu Horea Bedreag, Elena Sîrbu, Dorel Sandesc, Simona Ruxanda Dragan

**Affiliations:** 1 Doctoral School, “Victor Babes” University of Medicine and Pharmacy, E. Murgu Square, No. 2, 300041 Timisoara, Romania; 2 Department VI-Cardiology, “Victor Babes” University of Medicine and Pharmacy, 300041 Timisoara, Romania; 3 Research Centre of Timisoara Institute of Cardiovascular Diseases, “Victor Babes” University of Medicine and Pharmacy, 300041 Timisoara, Romania; 4 Department of Anesthesiology and Intensive Care, “Victor Babes” University of Medicine and Pharmacy, 300041 Timisoara, Romania; 5 “Pius Brinzeu” Emergency Clinical County Hospital, Bld Liviu Rebreanu, No. 156, 300723 Timisoara, Romania; 6 Department XVI, Medical Recovery, “Victor Babes” University of Medicine and Pharmacy, 300041 Timisoara, Romania; 7 Research Center for Assessment of Human Motion and Functionality and Disability, “Victor Babes” University of Medicine and Pharmacy, Eftimie Murgu Square, No. 2, 300041 Timisoara, Romania; 8 Department of Physical Therapy and Special Motricity, West University of Timisoara, 300223 Timisoara, Romania

**Keywords:** heart failure with reduced ejection fraction, telemedicine, digital health, remote monitoring, guideline-directed medical therapy, treatment adherence

## Abstract

**Background**: Many patients with heart failure with reduced ejection fraction (HFrEF) remain undertreated in routine practice. Delayed treatment intensification, poor adherence, and fragmented follow-up are common barriers. Low-cost digital support may help reduce this implementation gap. **Objective**: This study evaluated whether a simple digital support pathway was associated with better 6-month treatment adherence and guideline-directed medical therapy (GDMT) optimization in ambulatory patients with stable HFrEF. **Methods**: This single-center matched cohort study compared a prospective digital-support cohort with a historical usual-care cohort. The intervention combined smartphone-based telemanagement, home blood pressure and heart-rate reporting, daily weight surveillance, and scheduled video consultations. The co-primary endpoints were treatment adherence at 6 months and GDMT optimization, assessed by change in foundational HFrEF drug classes and by a prespecified exploratory GDMT optimization score. **Results**: After 1:1 propensity-score matching, 200 patients were included, with 100 patients in each cohort. Treatment adherence at 6 months was higher in the digital-support cohort than in usual care (82.0% vs. 64.0%, *p* = 0.004). The intervention cohort also had more frequent class addition, more dose escalation, a greater increase in foundational drug classes, and a larger improvement in GDMT optimization score (all *p* < 0.001). Heart failure hospitalization and the composite of heart failure hospitalization or all-cause death were less frequent in the digital-support cohort, but these clinical outcomes were exploratory. **Conclusions**: A pragmatic low-cost digital support pathway was associated with better adherence and more complete GDMT optimization in ambulatory patients with HFrEF. The findings support further prospective multicenter evaluation.

## 1. Introduction

Heart failure remains a major cause of morbidity, hospitalization, and death. HFrEF is the heart failure phenotype with the clearest evidence-based pharmacological framework. Current care is centered on four foundational drug classes: ARNI or ACEi/ARB, beta-blocker, MRA, and SGLT2 inhibitor [1,2,3,4,5]. Early initiation and progressive up-titration of these therapies are central goals of contemporary HFrEF management.

However, real-world delivery remains incomplete. Many patients do not receive all the recommended classes. Others remain on low doses or experience long delays between follow-up visits. This creates an implementation gap between guideline recommendations and routine outpatient care [6,7,8,9,10]. The problem is especially relevant in centers where outpatient capacity is limited and where frequent in-person reassessment is difficult.

The research gap is therefore practical rather than theoretical. The benefit of GDMT is well established, but the best low-cost method for maintaining adherence and accelerating optimization in daily care is less clear [11,12]. Many digital heart failure programs use dedicated platforms, complex monitoring systems, or specialized virtual teams. These models may be difficult to reproduce in older patients and in lower-resource clinical settings [13,14,15].

The motivation for the present study was to test a simpler approach. We focused on tools already available to most patients and clinics: smartphone communication, home blood pressure and heart-rate reporting, daily body-weight surveillance, and scheduled video follow-up. The intervention was designed to support clinicians in detecting intolerance, reinforcing adherence, and acting earlier on opportunities for GDMT intensification.

Based on this rationale, the present study evaluated a pragmatic low-cost digital support pathway in ambulatory patients with stable HFrEF. The objective was to assess whether this pathway was associated with better 6-month treatment adherence and GDMT optimization compared with matched usual care. Clinical event outcomes were also explored, but the study was not powered to establish definitive effects on hospitalization or mortality.

## 2. Materials and Methods

### 2.1. Study Design and Setting

This single-center matched cohort study evaluated a pragmatic, low-cost digital support pathway for ambulatory patients with HFrEF undergoing outpatient GDMT optimization over a 6-month follow-up period. The study included a prospective intervention cohort and a matched historical usual-care cohort from the immediately preceding institutional period. The study was designed as an exploratory implementation analysis rather than as a randomized efficacy trial.

Because the comparator group was historical, the design was intrinsically exposed to time-related bias. Secular changes in HFrEF management, clinician familiarity with contemporary GDMT, medication availability, outpatient workflow, or local hospitalization thresholds may have influenced the observed differences independently of the digital-support pathway. Propensity-score matching was used to improve measured baseline comparability, but it cannot remove residual confounding from unmeasured variables or from changes in practice over time.

The protocol was based on contemporary HFrEF treatment recommendations centered on the four foundational drug classes [2,3,5] and on evidence supporting structured remote support for closer follow-up and earlier treatment optimization [13,14,15].

### 2.2. Study Population

The study population consisted of consecutive adult patients aged 18 years or older with chronic stable HFrEF. HFrEF was defined by a left ventricular ejection fraction of 40% or less on echocardiography. Eligible patients had NYHA functional class II–III symptoms and active follow-up in a heart failure or cardiovascular rehabilitation program [4]. Patients were included only if they were clinically stable at enrollment, able to participate in home monitoring, and able to use smartphone-based communication independently or with caregiver support. This population was selected because evidence-based therapeutic targets for rapid GDMT implementation are most clearly established in HFrEF [3,16].

Patients were excluded when participation could be unsafe or when follow-up could not be interpreted reliably. Exclusion criteria were acute decompensated heart failure at screening, recent acute coronary syndrome, major cardiovascular instability, severe symptomatic hypotension, or bradyarrhythmia limiting GDMT titration. Patients were also excluded for advanced non-cardiac disease with limited life expectancy, severe musculoskeletal or neurological impairment preventing rehabilitation follow-up, major neuropsychiatric disease interfering with protocol adherence, uncontrolled thyroid dysfunction likely to confound symptoms or heart rate, or inability to participate in simple phone-based monitoring despite family support. These criteria were used to protect patient safety and to reduce major non-cardiac barriers to medication optimization and follow-up adherence.

### 2.3. Intervention Pathway

Patients in the intervention cohort were managed through a low-cost digital support pathway built on three components: smartphone-based telemanagement, home blood pressure and heart-rate reporting, and daily body-weight surveillance. The smartphone was used for bidirectional communication with the care team, adherence reinforcement, symptom reporting, confirmation of medication changes, and rapid escalation to medical review when warning signs appeared. Blood pressure, heart rate, and body weight were transmitted by phone call, text message, or WhatsApp message according to patient preference and digital ability.

Patients were instructed to report blood pressure and heart rate after resting in a seated position and to report body weight under similar daily conditions, preferably in the morning. However, the pathway did not use automated device-based data transfer or centralized calibration of home devices. Therefore, home blood pressure, heart rate, and body-weight values should be interpreted as pragmatic patient-reported monitoring data rather than objectively validated telemonitoring measurements.

All intervention patients had an initial in-person baseline assessment followed by scheduled video consultations at months 2, 4, and 6. Structured remote contacts were scheduled twice weekly during the first month, weekly during months 2 and 3, and every 2 weeks during months 4 to 6. Additional contacts were triggered by worsening dyspnea, edema, rapid weight gain, symptomatic hypotension, bradycardia, tachyarrhythmia symptoms, renal-function alerts, hyperkalemia, or suspected drug intolerance. Incoming data were reviewed by the heart failure/cardiovascular rehabilitation team. Medication changes were made only after physician review, and GDMT titration followed predefined safety checks for blood pressure, heart rate, congestion status, renal function, potassium, and patient-reported symptoms. The pathway was designed to reduce therapeutic inertia while remaining feasible for routine outpatient practice [13,16,17,18,19].

### 2.4. Usual-Care Cohort

The comparator cohort represented standard outpatient care before implementation of the digital support pathway. These patients received routine clinic-based follow-up, conventional rehabilitation counseling, and medication adjustment according to physician judgment. They did not receive structured remote surveillance, predefined smartphone-based contact, scheduled video consultations, or standardized home transmission of blood pressure, heart rate, and weight data. The comparator was selected to reflect the real-world pace of outpatient GDMT optimization before implementation of the digital pathway. Both cohorts were managed in the same institutional environment and by the same clinical service, but the historical design still allows residual confounding and secular changes in practice.

### 2.5. Baseline and Follow-Up Assessments

At baseline, patient-level data were extracted from institutional records and study follow-up forms. The database included age, sex, body mass index (BMI), HF etiology, comorbidities, NYHA class, systolic and diastolic blood pressure, resting heart rate, left ventricular ejection fraction, serum creatinine, estimated glomerular filtration rate, serum potassium, NT-proBNP, and current HF medication profile. Medication classes specifically recorded were ARNI or ACEi/ARB, beta-blocker, MRA, SGLT2 inhibitor, and loop diuretic. Follow-up reassessment at 6 months included the same clinical, laboratory, and treatment variables, together with hospitalizations, mortality, dose changes, discontinuations, and adherence metrics. These variables were selected because blood pressure, heart rate, renal function, potassium, congestion status, and natriuretic peptides guide safe HFrEF titration.

### 2.6. Definition of GDMT

For the purposes of this study, foundational GDMT for HFrEF was defined as prescription of the following four evidence-based therapeutic classes when not contraindicated: ARNI/ACEi/ARB, beta-blocker, MRA, and SGLT2 inhibitor [2,3,4]. Dose intensities for ARNI/ACEi/ARB, beta-blockers, and MRAs were categorized as absent, less than 50% of guideline target dose, or at least 50% of guideline target dose or maximally tolerated dose. SGLT2 inhibitor therapy was treated as prescribed or not prescribed because fixed-dose use is standard in routine practice. The GDMT optimization score used in this study was internally defined. It was prespecified for this analysis and should be interpreted as an exploratory implementation metric, not as a previously validated clinical endpoint.

The 0–7 structure was chosen to capture two complementary dimensions of GDMT implementation: breadth of foundational class coverage and partial achievement of dose intensity for therapies requiring titration. The ≥50% target-dose threshold was selected as a pragmatic marker of clinically meaningful dose implementation during a 6-month outpatient optimization period, while also allowing recognition of physician-documented maximally tolerated dosing in patients limited by blood pressure, renal function, potassium, heart rate, frailty, or symptoms.

### 2.7. Primary Endpoints

The study had two co-primary endpoints assessed at 6 months.

The first co-primary endpoint was treatment adherence at 6 months. Adherence was defined as persistent continuation of prescribed foundational HFrEF therapy without unadvised discontinuation and medication implementation corresponding to a proportion of days covered of at least 80%. This endpoint was selected because persistence and implementation are core dimensions of heart failure medication adherence, and non-adherence remains a major barrier to the real-world effectiveness of GDMT.

The second co-primary endpoint was GDMT optimization at 6 months. It was assessed in two complementary ways: change in the number of foundational HFrEF classes prescribed between baseline and month 6, and change in the internally defined GDMT optimization score ranging from 0 to 7. One point was assigned for each foundational class. One additional point was assigned for ARNI/ACEi/ARB, beta-blocker, and MRA when the patient reached at least 50% of the target dose or the maximally tolerated dose documented by the treating physician.

### 2.8. Secondary Endpoints

Secondary endpoints included prescription of at least 3 foundational classes at 6 months, prescription of all 4 foundational classes, any GDMT intensification during follow-up, time to first GDMT intensification, NYHA class change, NT-proBNP change, HF hospitalization, all-cause hospitalization, all-cause death, and the composite of HF hospitalization or all-cause death. Safety endpoints included symptomatic hypotension, bradycardia requiring treatment adjustment, worsening renal function, hyperkalemia, and drug discontinuation due to intolerance. Hospitalizations and deaths were identified from institutional medical records and structured follow-up contacts. Potential HF hospitalizations were reviewed against clinical documentation for congestion, HF-directed treatment, and discharge diagnosis. Safety events were extracted from clinical notes, laboratory results, and documented medication changes. Because the intervention cohort had more frequent contact with clinicians, detection and documentation bias were considered possible and were addressed in the limitations.

### 2.9. Matching Strategy

To improve comparability between cohorts, the usual-care cohort was matched 1:1 to the intervention cohort using nearest-neighbor propensity-score matching without replacement and with a caliper width of 0.2 standard deviations of the logit of the propensity score. Variables included in the propensity model were age, sex, LVEF, NYHA class, HF etiology, diabetes mellitus, atrial fibrillation, chronic kidney disease, systolic blood pressure, heart rate, baseline NT-proBNP, baseline number of GDMT classes, and recent HF hospitalization. Covariate balance after matching was assessed using standardized mean differences, with values below 0.10 considered acceptable. Table 1 reports the post-matching balance for all variables included in the model. A Love plot of post-match standardized mean differences was added as Supplementary Figure S1 to improve transparency.

### 2.10. Statistical Analysis

Data entry and cleaning were performed using Microsoft Excel 2016 (Microsoft Corp., Redmond, WA, USA) [20]. Statistical analyses were conducted using IBM SPSS Statistics version 26 (IBM Corp., Armonk, NY, USA) [21] and R version 4.3.3 (R Foundation for Statistical Computing, Vienna, Austria) [22].

Continuous variables were summarized as mean ± standard deviation or median with interquartile range, depending on distribution, and categorical variables as counts and percentages. Between-group comparisons were performed using Student’s t test or the Mann–Whitney U test for continuous variables and the chi-square test or Fisher’s exact test for categorical variables. The 6-month adherence endpoint was analyzed using logistic regression adjusted for baseline covariates and reported as odds ratios with 95% confidence intervals. Change in GDMT optimization score was analyzed using analysis of covariance with baseline score included as a covariate. Time-to-event secondary outcomes were evaluated with Kaplan–Meier curves and Cox proportional hazards models. A two-sided *p* value below 0.05 was considered statistically significant.

Because the study used co-primary endpoints, hypothesis testing was prespecified in hierarchical order. Treatment adherence was tested first, and GDMT optimization was tested second only if the first endpoint met statistical significance. Missingness was examined before imputation. Multiple imputation by chained equations was performed under a missing-at-random assumption when variables required for adjusted models were incomplete. The imputation model included baseline covariates, cohort assignment, treatment variables, adherence status, 6-month GDMT score, hospitalization outcomes, and vital status. Twenty imputed datasets were generated, and pooled estimates were compared with complete-case analyses as sensitivity analyses. Clinical event analyses were considered exploratory because of the short follow-up and limited event numbers.

No formal adjustment for calendar time was performed because cohort assignment and treatment period were closely linked. For this reason, all between-group comparisons involving hospitalization, death, or the composite clinical endpoint were interpreted as exploratory associations and not as causal estimates of intervention efficacy.

### 2.11. Ethical Considerations

The study was conducted in accordance with the Declaration of Helsinki [23] and received approval from the Ethics Committee of the University of Medicine and Pharmacy “Victor Babeș” Timișoara (approval no. 111/2020, revised 12 May 2026) and from the Ethics Committee of the Institute of Cardiovascular Diseases, Timișoara (approval no. 1454/19 February 2026). The earlier institutional approval covered the retrospective use of de-identified clinical data from the historical usual-care cohort. For this retrospective component, individual informed consent was waived by the ethics committee because data were extracted from existing records and anonymized before analysis. The 2026 approval covered the prospective digital-support pathway and the final matched cohort analysis. All patients included in the prospective digital-support cohort received study information and signed informed consent before participation. Refusal to participate or withdrawal from prospective follow-up did not affect the quality of care received. Data handling complied with the General Data Protection Regulation (EU 2016/679) [24].

## 3. Results

### 3.1. Study Flow and Baseline Characteristics

**Figure 1:**
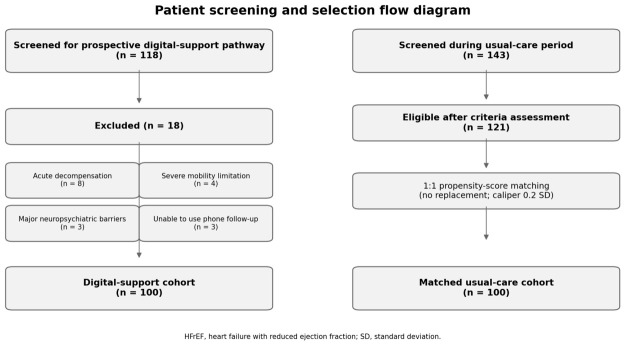
Study flow of the cohorts. The flow diagram summarizes screening, exclusion, inclusion, and propensity-score matching for the digital-support and usual-care cohorts.

For the prospective digital-support pathway, 118 patients were screened. Eighteen were excluded because of acute decompensation (n = 8), severe mobility limitation (n = 4), major neuropsychiatric barriers (n = 3), or inability to participate in simple phone-based follow-up (n = 3). The final intervention cohort included 100 patients. In the usual-care period, 143 patients were screened, 121 fulfilled eligibility criteria, and 100 were retained after 1:1 propensity-score matching. The study flow is presented in Figure 1.

After matching, baseline characteristics were well balanced between groups. All post-match standardized mean differences were below 0.10. The cohorts were comparable in age, sex distribution, HF etiology, symptom burden, renal function, natriuretic peptide levels, and baseline treatment intensity. Baseline characteristics are summarized in Table 1.

### 3.2. Co-Primary Endpoints: Treatment Adherence and GDMT Optimization

At 6 months, treatment adherence was higher in the digital-support cohort than in the usual-care cohort (82.0% vs. 64.0%, *p* = 0.004). Persistent continuation of prescribed foundational therapy was also more frequent in the intervention group (86.0% vs. 70.0%, *p* = 0.007). A proportion of days covered of at least 80% was achieved more often in the digital-support cohort (79.0% vs. 61.0%, *p* = 0.006). In adjusted logistic regression, participation in the digital-support pathway was associated with higher odds of 6-month treatment adherence (OR 2.56, 95% CI 1.39–4.72, *p* = 0.002).

By month 6, patients managed through the digital-support pathway were more likely to receive at least 3 foundational HFrEF classes (88.0% vs. 58.0%, *p* < 0.001) and all 4 foundational classes (52.0% vs. 28.0%, *p* < 0.001). The mean number of foundational classes at 6 months was 3.39 ± 0.74 in the digital-support cohort and 2.77 ± 0.94 in the usual-care cohort (*p* < 0.001). Median time to first treatment intensification was shorter in the digital-support cohort (24 [14–43] days vs. 63 [34–103] days, *p* < 0.001).

**Figure 2:**
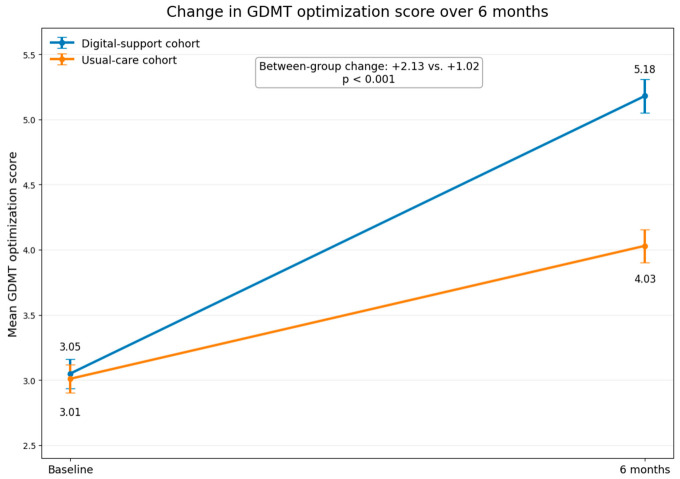
Change in GDMT optimization score from baseline to 6 months. The figure shows mean GDMT optimization score values with standard-error bars in the matched cohorts. Baseline values were similar between groups. At 6 months, the digital-support cohort had a greater increase in score (+2.13 ± 1.20 vs. +1.02 ± 1.11; *p* < 0.001). GDMT, guideline-directed medical therapy; SE, standard error.

The co-primary endpoints are summarized in Table 2, while the between-group change in mean GDMT optimization score from baseline to 6 months is shown in Figure 2.

At 6 months, clinical status showed exploratory differences in favor of the digital-support cohort. Improvement by at least one NYHA class was observed in 47.0% of patients in the digital-support group and in 26.0% of patients in usual care (*p* = 0.002). A relative NT-proBNP reduction of at least 30% was also more frequent in the digital-support cohort (44.0% vs. 27.0%, *p* = 0.012). HF hospitalization occurred in 14.0% of digitally supported patients and in 26.0% of usual-care patients (*p* = 0.033). The composite endpoint of HF hospitalization or all-cause death occurred in 18.0% versus 31.0%, respectively (*p* = 0.034). Kaplan–Meier analysis showed a lower cumulative risk in the digital-support cohort, with an estimated hazard ratio of 0.54 (95% CI 0.30–0.96, *p* = 0.036). These event analyses should be interpreted as hypothesis-generating because of the non-randomized design, short follow-up, and limited number of events.

No significant between-group differences were observed for symptomatic hypotension, bradycardia requiring treatment adjustment, worsening renal function, hyperkalemia, or drug discontinuation due to intolerance. This suggests that more intensive titration was not associated with an evident short-term safety penalty within the limits of the available sample size.

Secondary outcomes and tolerability are presented in Table 3.

**Figure 3:**
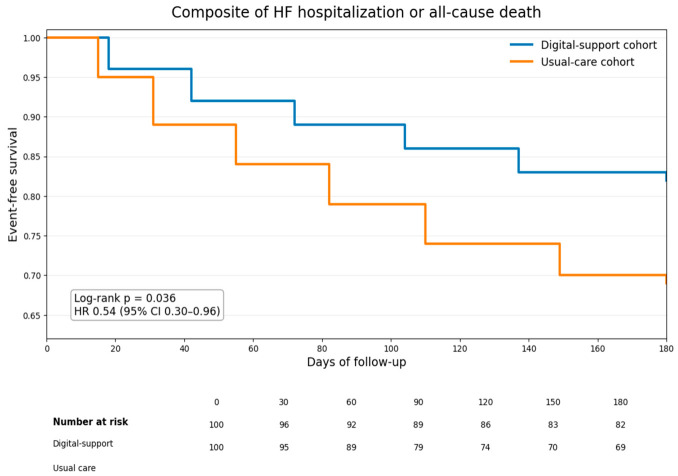
Kaplan–Meier estimate for the exploratory composite endpoint of HF hospitalization or all-cause death. The *p*-value from the log-rank test is shown on the graph, and the number-at-risk table is presented below the curves. HR, hazard ratio; CI, confidence interval; HF, heart failure.

The time-to-event analysis for the composite of HF hospitalization or all-cause death is shown in Figure 3.

## 4. Discussion

### 4.1. Main Findings and Clinical Interpretation

In this study, a pragmatic low-cost digital support pathway was associated with better 6-month treatment adherence and more complete GDMT implementation in ambulatory patients with HFrEF. The intervention cohort had higher persistence on prescribed therapy, more frequent class addition and dose escalation, and a greater proportion of patients receiving three or four foundational drug classes by month 6. These findings are clinically plausible because contemporary HFrEF care often fails at the implementation stage rather than at the evidence-generation stage. Current expert pathways emphasize early use of the four foundational drug classes and timely titration to target or maximally tolerated doses [5], while real-world reports continue to show persistent GDMT underuse [6,7,8,9,10].

The findings are consistent with recent digital-management literature, but they should not be interpreted as proof of causality. The ADMINISTER randomized trial showed that a digital consult strategy improved GDMT optimization over 12 weeks, and the ESC digital-health consensus statement emphasizes that remote tools are most useful when they are connected to active clinical review and treatment optimization [13,25,26,27,28]. In the present study, the likely value of the pathway was not the smartphone itself. It was a structured opportunity to review symptoms, reinforce adherence, detect intolerance, and act earlier on treatment opportunities.

The practical strength of the model is its simplicity. It did not require implantable sensors, dedicated commercial platforms, or high digital literacy. It relied on tools already familiar to many patients and clinics: phone communication, text messages, home blood pressure and heart-rate reporting, daily weight surveillance, and scheduled video follow-up. This may be relevant for older patients and for clinical systems where scalable, low-cost interventions are needed [15,29,30,31].

### 4.2. Comparison with Previous Studies on GDMT Optimization

The present findings are broadly consistent with the recent literature showing that structured remote support can improve GDMT implementation in HFrEF. In the ADMINISTER randomized trial, a digital consult strategy produced a greater 12-week improvement in GDMT score than usual care [28]. Recent consensus and review papers also emphasize that digital interventions appear most useful when they are designed around medication optimization, not only around passive education or symptom recording [13,32].

Our results also support the idea that the intensity and function of follow-up may matter more than the technology platform itself. Repeated low-friction contact can help clinicians identify opportunities for titration, reinforce medication persistence, and respond earlier to tolerability problems. This interpretation is aligned with studies of remote management platforms and virtual-care strategies that have reported improved GDMT optimization when remote data were linked to clinical action [33,34,35].

At the same time, the present study differs from many prior digital HF reports because it intentionally used a minimal-tech structure [23,36]. This makes the model less technologically ambitious but potentially easier to transfer to routine outpatient practice. The trade-off is that the pathway requires disciplined clinical workflow, clear safety thresholds, and physician availability for medication decisions. These operational elements should be standardized in future studies before the model is implemented more widely.

### 4.3. Clinical Outcomes Beyond GDMT Optimization

Although the present study was not designed or powered for hard clinical events, the exploratory event pattern favored the digital-support pathway. Lower rates of HF hospitalization and of the composite endpoint of HF hospitalization or all-cause death are biologically plausible in the context of better adherence and more complete GDMT implementation. However, the interpretation must remain cautious. A small single-center non-randomized study with 6 months of follow-up can overestimate event differences, and residual confounding may persist despite propensity-score matching.

The direction of the hospitalization signal is consistent with broader heart failure literature. A recent comprehensive meta-analysis reported that remote patient monitoring was associated with lower mortality and fewer first HF hospitalizations, especially when programs included self-management, education, and video communication [11]. Exercise-based cardiac rehabilitation also improves quality of life and reduces hospital admission in adults with heart failure, although mortality effects are less certain over shorter follow-up [8,37]. In this context, the present event findings should be considered supportive and hypothesis-generating rather than definitive evidence of event reduction.

One possible explanation is that the intervention improved intermediate steps that occur before clinical decompensation. Better medication persistence, earlier class addition, earlier dose escalation, and repeated surveillance may allow earlier correction of congestion, intolerance, or adherence problems. This mechanism is plausible, but it remains inferential. The current data demonstrate association, not direct proof that the pathway caused fewer events.

The 6-month horizon was appropriate for evaluating early adherence, early medication persistence, and initial GDMT selection. It was less appropriate for assessing durability. Persistence of adherence, long-term dose maintenance, reverse remodeling, recurrent hospitalization, and mortality require longer follow-up. Therefore, the clinical event signal observed here should be viewed as an early implementation signal rather than evidence of sustained prognostic benefit.

### 4.4. Limitations and Future Perspectives

Several limitations should be acknowledged. First, this was a single-center study with a limited sample size, so the findings may not generalize to other health systems, rural settings, or patients with lower digital access. Second, the usual-care cohort was historical. This is an important limitation because time bias may persist even after propensity-score matching. Changes in clinician behavior, institutional workflow, GDMT availability, background guideline uptake, rehabilitation practice, or hospitalization thresholds may have contributed to the observed differences independently of the intervention. Third, follow-up was limited to 6 months. This duration is adequate for assessing early adherence, treatment persistence, and GDMT dose selection, but it is insufficient for conclusions about long-term sustainability, durable clinical benefit, recurrent hospitalization, or mortality.

Additional limitations relate to endpoint construction and ascertainment. Medication adherence is difficult to measure perfectly in routine care and may reflect persistence, implementation, documentation quality, and follow-up intensity. The GDMT optimization score was internally defined and has not been externally validated. It should therefore be interpreted as an exploratory implementation metric. Event ascertainment may also be affected by detection bias, because intervention patients had more frequent contact with clinicians. Finally, treatment optimization can be limited by hypotension, renal dysfunction, hyperkalemia, bradycardia, frailty, and patient preference, so medication counts do not fully capture therapeutic opportunity.

Future work should move toward prospective, multicenter, randomized or cluster-randomized designs with standardized telemanagement protocols and prespecified implementation endpoints. A logical next step would be to test this minimal-tech model in a wider trial focused on medication persistence, time to GDMT intensification, class achievement, tolerability, cost-effectiveness, patient acceptability, and equity of access. Subgroup analyses in older adults, patients with caregiver-assisted digital use, and patients with limited digital literacy would be especially important [12,38,39,40,41,42].

More complex digital rehabilitation technologies, including sensor-based monitoring and assistive devices, may become relevant in future programs. They were not evaluated in the present study and should be investigated separately in dedicated feasibility and efficacy trials.

Future work should move toward prospective, multicenter, randomized or cluster-randomized designs with standardized telemanagement protocols and prespecified implementation endpoints. A logical next step would be to test this minimal-tech model in a wider trial focused on medication persistence, time to GDMT intensification, achievement of foundational drug classes, tolerability, patient acceptability, cost-effectiveness, and equity of access. Subgroup analyses in older adults, patients requiring caregiver-assisted digital communication, and patients with limited digital literacy would be especially important. More complex digital rehabilitation technologies, including sensor-based monitoring and automated data transmission, were not evaluated in the present study and should be investigated separately in dedicated feasibility and efficacy trials.

## 5. Conclusions

In this exploratory single-center matched cohort study, a pragmatic low-cost digital support pathway was associated with better 6-month treatment adherence and more complete GDMT optimization in ambulatory patients with stable HFrEF. The pathway used simple, widely available tools to support repeated monitoring, adherence reinforcement, and earlier therapeutic review. Clinical event findings favored the digital-support cohort but should be interpreted as hypothesis-generating because of the historical comparator, short follow-up, and limited sample size. Prospective multicenter trials are needed to confirm whether this minimal-tech model can improve durable GDMT implementation and clinical outcomes in routine heart failure care.

## Figures and Tables

**Table 1:** Baseline characteristics of the matched cohort.

Variable	Digital-Support Cohort (n = 100), Value	Usual-Care Cohort (n = 100), Value	*p*-Value	SMD
Age, years	67.4 ± 10.2	68.1 ± 9.8	0.620	0.07
Male sex, n (%)	72 (72.0)	70 (70.0)	0.750	0.04
Ischemic HF etiology, n (%)	58 (58.0)	60 (60.0)	0.770	0.04
NYHA class III, n (%)	46 (46.0)	44 (44.0)	0.780	0.04
LVEF, %	31.3 ± 5.8	31.0 ± 6.0	0.710	0.05
Diabetes mellitus, n (%)	41 (41.0)	39 (39.0)	0.770	0.04
Atrial fibrillation, n (%)	29 (29.0)	27 (27.0)	0.750	0.04
Chronic kidney disease, n (%)	31 (31.0)	30 (30.0)	0.880	0.02
Recent HF hospitalization, n (%)	48 (48.0)	47 (47.0)	0.890	0.02
Systolic blood pressure, mmHg	118 ± 14	119 ± 15	0.680	0.07
Resting heart rate, bpm	74 ± 11	75 ± 10	0.520	0.09
Serum creatinine, mg/dL	1.14 ± 0.32	1.16 ± 0.35	0.670	0.06
NT-proBNP, pg/mL	1650 [980–2820]	1710 [1010–2910]	0.610	0.05
Number of foundational GDMT classes at baseline	2.34 ± 0.91	2.31 ± 0.88	0.820	0.03
GDMT optimization score at baseline	3.05 ± 1.12	3.01 ± 1.09	0.790	0.04

Values are presented as mean ± SD, median [IQR], or n (%), as appropriate. For example, 67.4 ± 10.2 indicates mean ± SD. Statistical significance was defined as a two-sided *p* < 0.05. Post-match balance was considered acceptable when SMD < 0.10. LCL and UCL refer to the 95% lower and upper confidence limits for continuous-variable means and were added to the supplementary balance output to avoid overloading the main baseline table. SMD, standardized mean difference; HF, heart failure; NYHA, New York Heart Association; LVEF, left ventricular ejection fraction; GDMT, guideline-directed medical therapy.

**Table 2:** Co-primary endpoints and treatment implementation at 6 months.

Variable	Digital-Support Cohort (n = 100), n (%) or Value	Usual-Care Cohort (n = 100), n (%) or Value	*p*-Value
Adherence outcomes			
Treatment adherence at 6 months, n (%)	82 (82.0)	64 (64.0)	0.004
Persistent continuation of prescribed foundational therapy, n (%)	86 (86.0)	70 (70.0)	0.007
Proportion of days covered ≥80%, n (%)	79 (79.0)	61 (61.0)	0.006
Therapy intensification			
Any class addition during follow-up, n (%)	63 (63.0)	34 (34.0)	<0.001
Any dose escalation during follow-up, n (%)	71 (71.0)	39 (39.0)	<0.001
6-month class implementation			
ARNI/ACEi/ARB prescribed at 6 months, n (%)	91 (91.0)	80 (80.0)	0.026
Beta-blocker prescribed at 6 months, n (%)	94 (94.0)	85 (85.0)	0.034
MRA prescribed at 6 months, n (%)	78 (78.0)	60 (60.0)	0.006
SGLT2 inhibitor prescribed at 6 months, n (%)	76 (76.0)	52 (52.0)	<0.001
Patients on ≥3 foundational classes at 6 months, n (%)	88 (88.0)	58 (58.0)	<0.001
Patients on 4 foundational classes at 6 months, n (%)	52 (52.0)	28 (28.0)	<0.001
GDMT score and timing			
Number of foundational classes at 6 months	3.39 ± 0.74	2.77 ± 0.94	<0.001
Change in number of foundational classes	+1.05 ± 0.82	+0.46 ± 0.77	<0.001
GDMT optimization score at 6 months	5.18 ± 1.29	4.03 ± 1.26	<0.001
Change in GDMT optimization score	+2.13 ± 1.20	+1.02 ± 1.11	<0.001
Time to first treatment intensification, days	24 [14–43]	63 [34–103]	<0.001

Categorical variables are presented as n (%). Continuous normally distributed variables are presented as mean ± SD, and skewed variables as median [IQR]. Statistical significance was defined as a two-sided *p* < 0.05. The GDMT optimization score ranged from 0 to 7 and was used as an internally defined exploratory implementation metric. ARNI, angiotensin receptor-neprilysin inhibitor; ACEi, angiotensin-converting enzyme inhibitor; ARB, angiotensin receptor blocker; MRA, mineralocorticoid receptor antagonist; SGLT2, sodium-glucose cotransporter-2.

**Table 3:** Secondary outcomes and safety at 6 months.

Variable	Digital-Support Cohort (n = 100), n (%) or Median [IQR]	Usual-Care Cohort (n = 100), n (%) or Median [IQR]	*p*-Value
Improvement by at least one NYHA class, n (%)	47 (47.0)	26 (26.0)	0.002
NT-proBNP at 6 months, pg/mL	1190 [650–2140]	1450 [820–2450]	0.041
Relative NT-proBNP reduction ≥30%, n (%)	44 (44.0)	27 (27.0)	0.012
HF hospitalization, n (%)	14 (14.0)	26 (26.0)	0.033
All-cause hospitalization, n (%)	20 (20.0)	31 (31.0)	0.072
All-cause death, n (%)	5 (5.0)	8 (8.0)	0.390
Composite of HF hospitalization or all-cause death, n (%)	18 (18.0)	31 (31.0)	0.034
Symptomatic hypotension, n (%)	11 (11.0)	8 (8.0)	0.470
Bradycardia requiring treatment adjustment, n (%)	6 (6.0)	4 (4.0)	0.520
Worsening renal function, n (%)	9 (9.0)	7 (7.0)	0.600
Hyperkalemia, n (%)	7 (7.0)	5 (5.0)	0.550
Drug discontinuation due to intolerance, n (%)	8 (8.0)	10 (10.0)	0.620

Categorical variables are presented as n (%), and NT-proBNP is presented as median [IQR]. Statistical significance was defined as a two-sided *p* < 0.05. Worsening renal function was defined as a clinically relevant rise in serum creatinine prompting treatment reassessment. Hyperkalemia was defined according to local clinical reporting thresholds during follow-up. NYHA, New York Heart Association; NT-proBNP, N-terminal pro-B-type natriuretic peptide; HF, heart failure.

## Data Availability

The minimal de-identified dataset supporting the main findings of this article is provided as Supplementary File for internal editorial evaluation. Additional data are available from the corresponding author upon reasonable request, subject to institutional approval and applicable GDPR restrictions.

## Supplementary Materials

The following supporting information can be downloaded at: https://www.mdpi.com/article/10.3390/healthcare14121675/s1, Supplementary Figure S1. Love Plot: Values are presented as mean ± standard deviation, median [interquartile range], or n (%), as appropriate. Statistical sig-nificance was defined as a two-sided *p* < 0.05. Post-match covariate balance was con-sidered acceptable when SMD < 0.10. This supplementary figure provides the full post-matching balance assessment for the variables included in the propensity-score model. SMD, standardized mean difference; HF, heart failure; NYHA, New York Heart Association; LVEF, left ventricular ejection fraction; GDMT, guideline-directed medical therapy; NT-proBNP, N-terminal pro-B-type natriuretic peptide.

## Abbreviations

ACEi	Angiotensin-converting enzyme inhibitor
AF	Atrial fibrillation
ARB	Angiotensin receptor blocker
ARNI	Angiotensin receptor-neprilysin inhibitor
BMI	Body mass index
BP	Blood pressure
CI	Confidence interval
CKD	Chronic kidney disease
eGFR	Estimated glomerular filtration rate
GDMT	Guideline-directed medical therapy
HF	Heart failure
HFrEF	Heart failure with reduced ejection fraction
HR	Hazard ratio or heart rate, according to context
IQR	Interquartile range
LVEF	Left ventricular ejection fraction
MRA	Mineralocorticoid receptor antagonist
NT-proBNP	N-terminal pro-B-type natriuretic peptide
NYHA	New York Heart Association
OR	Odds ratio
SGLT2i	Sodium-glucose cotransporter-2 inhibitor
SMD	Standardized mean difference
