# Supplementary material for: A Pragmatic Low-Cost Digital Support Pathway for GDMT Optimization in Ambulatory HFrEF: An Exploratory 6-Month Matched Cohort Study
Source: Healthcare (Basel). 2026 Jun 12;14(12):1675. doi: 10.3390/healthcare14121675 (PMC13300278; doi:10.3390/healthcare14121675)
Supplement: Supplementary file 1 [file healthcare-14-01675-s001.zip › healthcare-4311938-supplementary.pdf]

Supplementary Figure S1 Love Plot:

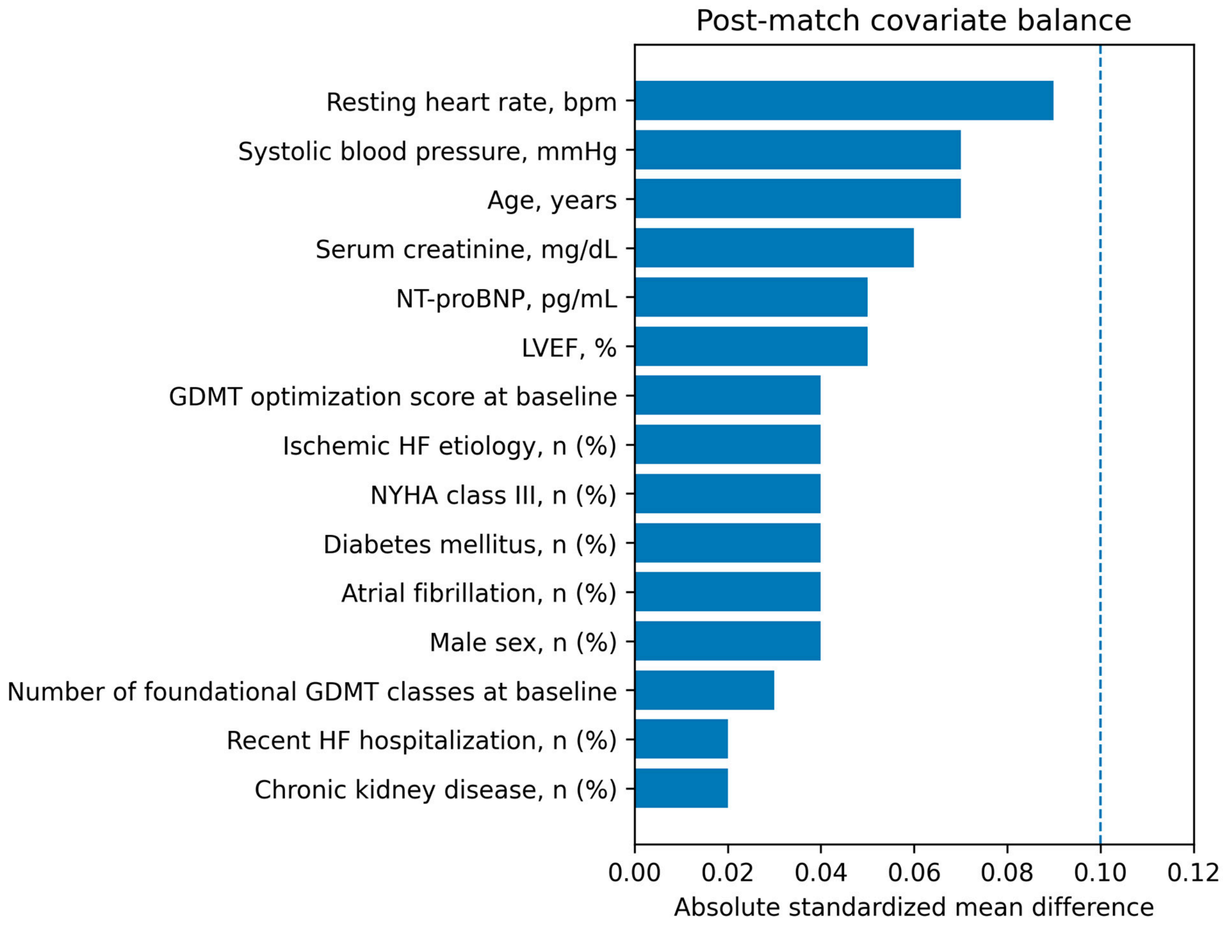

Values are presented as mean ± standard deviation, median [interquartile range], or n (%), as appropriate. Statistical significance was defined as a two-sided p < 0.05. Post-match covariate balance was considered acceptable when SMD < 0.10. This supplementary figure provides the full post-matching balance assessment for the variables included in the propensity-score model. SMD, standardized mean difference; HF, heart failure; NYHA, New York Heart Association; LVEF, left ventricular ejection fraction; GDMT, guideline-directed medical therapy; NT-proBNP, N-terminal pro-B-type natriuretic peptide.
